# Estimating transmission parameters from stochastic epidemic models using survival analysis techniques

**DOI:** 10.1177/09622802261468021

**Published:** 2026-07-15

**Authors:** Hein Putter, Chengyuan Lu, Jacco Wallinga

**Affiliations:** 1 4501 Leiden University Medical Center, Leiden, The Netherlands; 2 Department of Mathematics, Leiden University, Leiden, The Netherlands; 3 National Institute for Public Health and the Environment, Bilthoven, The Netherlands

**Keywords:** Compartmental epidemic models, counting processes, additive hazards, proportional hazards, Poisson models

## Abstract

Compartmental models based on ordinary differential equations quantifying the interactions between susceptible, infectious and recovered individuals within a population have played an important role in infectious disease modelling. The aim of the present paper is to explain the link between stochastic epidemic models based on the susceptible-infectious-recovered (SIR) model and methods from survival analysis. We illustrate how standard software for survival analysis in the statistical language R can be used to estimate pivotal parameters in the stochastic SIR model in the very much idealized situation where the epidemic is completely observed. Extensions incorporating interventions, age structure and heterogeneity are explored and illustrated.

## 1. Introduction

During the coronavirus disease 2019 (COVID-19) pandemic, compartmental epidemic transmission models have been crucial in informing infection control policies, making forecasts, exploring scenario’s and evaluating interventions. These compartmental epidemic models are typically described by ordinary differential equations (ODEs). The most famous representative of these models is quantifying the interactions between susceptible (S), infectious (I) and recovered (R) individuals within a population, the SIR model.^1–3^ Fitting such models to observations, with the aim of estimating pivotal quantities like transmission parameters, is challenging. Approaches to model fitting and parameter estimation that are frequently used include approximate Bayesian computation,^4,5^ iterated filtering,^6^ and sequential Monte Carlo or filtering.^7,8^ These methods are all computationally very intensive. For the purpose of informing policy, fast and explicit estimators that are statistically efficient, such as maximum likelihood estimators (MLEs), are of high interest. So far, MLE estimates are available for idealized epidemic models such as SIR and idealized data, assuming that the transmission rate is constant.^9^

Here we suggest the use of inferential approaches that are developed and used in survival analysis. These estimators rely on a coherent inferential framework (often maximum likelihood or partial likelihood), often we can obtain explicit estimators, and otherwise efficient algorithms have been developed and are available in standard statistical software. Most of these estimators allow for very flexible model assumptions, they can allow for a much broader ranges of transmission models than the very idealized SIR model.

As the statistical field of survival analysis is developing recently to include more general model structures, not all of these recent developments have been used in the field of infectious disease modelling.

The aim of this work is to develop new approaches to infer time-constant or time-varying transmission rates by building on the available work in the field of survival analysis. We first illustrate the basic ideas and terminology and notation in a highly idealized setting, with idealized data where all events in a population are observed, and an idealized stochastic epidemic model where all individuals are similar, except for their infection history, and transmission rate is constant. In this setting we can derive an explicit MLE estimator that we will use as a benchmark. We will show how time-varying transmission rates can be inferred and tests for constancy can be performed, and we will relax assumptions with respect to the underlying epidemic model, allowing for differences between groups of individuals and allowing for differences between individuals, additive terms such as import of infection, and multiplicative terms such as control measures that affect transmission rate. We will also suggest approaches to relax the assumptions made about the idealized data and allow for unobserved events, incompletely observed events and binned data. We will illustrate our approach using hands-on examples with R code, available online at https://github.com/survival-lumc/SIRsurvival. The scope of our approach includes compartmental models as used in, for example, the COVID-19 pandemic.

We first introduce the deterministic and stochastic SIR model, and review maximum likelihood estimation in this model, in particular the work of Becker & Britton.^9^ The central contribution of the paper is in Subsection 2.3, where we introduce *long format survival data* that allows standard techniques for survival analysis to estimate time-constant and time-varying rates, and to graphically and formally test whether the transmission rate is constant. Section 3 is devoted to illustrating how to use standard survival analysis techniques on this data format to estimate transmission rates, constant over time or varying over time, in an idealized setting where all infections are completely observed. In Section 4 we relax assumptions about the underlying epidemic models, to include an assessment of the effects of interventions, population structure and observed and unobserved heterogeneity in susceptibility. In Section 5 we relax the assumption of a completely and exactly observed epidemic and show how more realistic data structures, such as daily reports of the numbers of new infections, can be used as a basis to create synthetic long-format data from the available data. Section 6 contains a re-analysis of an outbreak of H1N1 influenza, highlighting how our methods may provide additional insight. We end with a discussion in Section 7.

## 2. The SIR model and a useful data format

### 2.1. Deterministic SIR model

The SIR model is a system of differential equations, describing the evolution over time of an infectious disease. Defining $\bar{S}(t)$ , $\bar{I}(t)$ and $\bar{R}(t)$ to be the proportion of SIR individuals in a closed population, the system of ODE is defined as^2^:

(1) $$\begin{array}{rl} \frac{d\bar{S}}{dt} & =-\beta \bar{S}(t)\bar{I}(t),\\ \frac{d\bar{I}}{dt} & =\beta \bar{S}(t)\bar{I}(t)-\gamma \bar{I}(t),\\ \frac{d\bar{R}}{dt} & =\gamma \bar{I}(t). \end{array}$$

The idea behind Equation (1) is that interactions between susceptible and infectious individuals leading to a new infection occur with a rate quantified by a transmission parameter β . In case of an infection the proportion of susceptibles decreases and the proportion of infectious individuals increases by the same amount. Infectious individuals recover with a rate quantified by the recovery parameter γ . This class of models is often referred to as *compartmental models*, since it describes the interactions of units from different compartments. Apart from describing the spread of an infection through a population, compartmental models have been used in many other fields of application. Among the many examples we mention models describing the interaction of HIV and CD4+ T-cells within humans,^10^ predator-prey models in biology^11^ and economics,^12^ and pharmacodynamics.^13^ Many extensions of this simple SIR model have been developed, but the simple SIR model has proved to be remarkably robust and we will stick with the simple SIR model for most of the paper.

**Figure 1.**
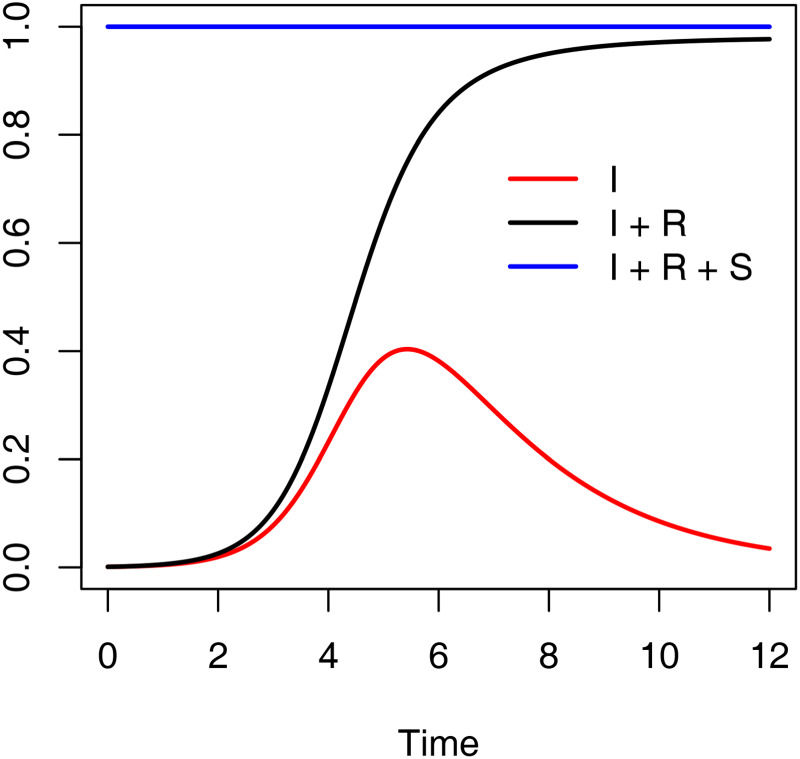
Stacked plot showing the proportion of susceptible, infected and recovered individuals over time in the susceptible-infectious-recovered (SIR) model; β=2 , γ=0.5 .

Figure 1 shows the development of the epidemic over time with parameter values β=2 , γ=0.5 and initial condition $\bar{I}(0)=0.001$ , in the form of a stacked plot. The lowest curve shows the proportion of infected individuals. The distance between the lowest curve and the one directly above that represents the proportion of recovered individuals. The sum of these two is the proportion of individuals that have got infected over time (recovered or not). The remainder is the proportion of susceptible individuals.

### 2.2. Stochastic SIR model

The deterministic SIR model can be thought of as a ‘law of large numbers’ limit of a stochastic system consisting of a large number of individuals, each of which has a rate of transitioning between states (up to a small term due to the covariance between I and S , see Isham^14^). The epidemic starts at time t=0 with I(0) , S(0) and R(0) individuals that are infected, susceptible and recovered, respectively, for a total number of n=S(0)+I(0)+R(0) individuals. Because the system is closed, we in fact have n=S(t)+I(t)+R(t) for all t . We again use bars for the proportions $\bar{S}(t)=S(t)/n$ , $\bar{I}(t)=I(t)/n$ and $\bar{R}(t)=R(t)/n$ of susceptibles, infecteds and recovered individuals, respectively.

Becker and Britton^9^ discuss maximum likelihood estimation under complete observation. Let N(t)=S(0)−S(t) be the number of individuals infected in (0,t] . This is a counting process, as well as R(t) , which is the number of individuals that recovered in (0,t] , if R(0)=0 . If $H_{t}$ is the σ -algebra generated by the history {S(u),I(u);0≤u<t} , then the epidemic can be expressed in terms of the rates of the counting processes N(t) and R(t) , ignoring terms of o(dt) , by

(2) $$\begin{array}{rl} P(dN(t)=1,dR(t)=0\;|\;H_{t}) & =\beta S(t)\bar{I}(t)dt,\\ P(dN(t)=0,dR(t)=1\;|\;H_{t}) & =\gamma I(t)dt,\\ P(dN(t)=0,dR(t)=0\;|\;H_{t}) & =1-\{\beta S(t)\bar{I}(t)-\gamma I(t)\}dt. \end{array}$$

At some finite (random) time τ all infectious individuals will have recovered and the epidemic is over. Increasing β leads to higher infection rates, while lower γ leads to a longer time being infected and thus more opportunity to infect others. The ratio β/γ is known as the *basic reproduction number* $R_{0}$ , the average number of infections caused by one typical infectious individual in a completely susceptible environment, which in our example equals 4.

**Figure 2.**
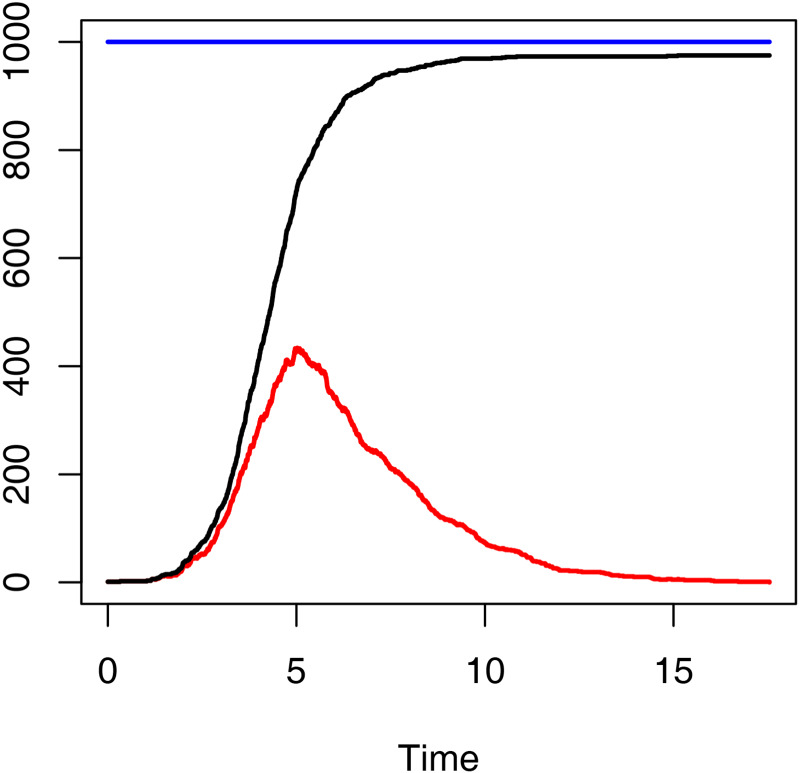
Stacked plot showing the number of susceptible, infected and recovered individuals over time from a stochastic susceptible-infectious-recovered (SIR) model.

**Figure 3.**
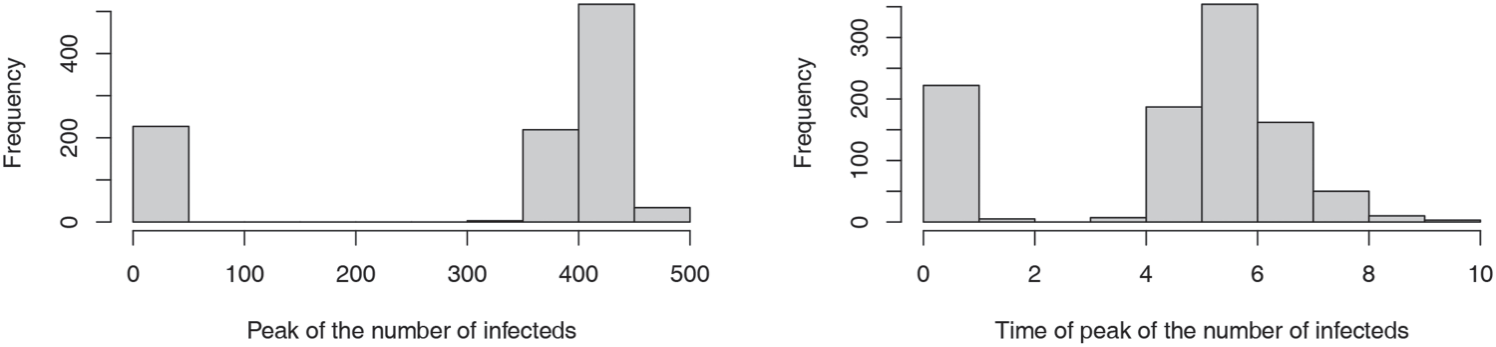
Results of 1000 simulated susceptible-infectious-recovered (SIR) epidemics. (a) Peak of the number of infecteds, (b) time of peak of the number of infecteds.

Figure 2 shows one realization of the stochastic process, with the same parameters as before ( β=2 , γ=0.5 ), a population size of 1000, and a single infectious individual at t=0 . A total of 25 subjects have remained uninfected. It looks very much like the deterministic system, but with, on average, a slight delay in the time of peak prevalence. Note, however, that the system is now stochastic. With another random seed we would obtain a different epidemic. Occasionally the epidemic might just not start off, because by chance the early infected individuals recover before they managed to infect new individuals. Figure 3 shows a histogram of (a) the peaks in number of infecteds and (b) the time at which they occurred in 1000 simulated epidemics, with the same parameters as before, a population size of 1000 and one initial infected individual.

We see that in about 23% of the simulated epidemics, only a very limited number of individuals (here at most five, including the first infected individual) got infected. This coincides with mathematical theory saying that with one initial infectious individual the probability of the epidemic not spreading to a large part of the population equals $1/R_{0}$ .^3^

Becker and Britton^9^ derived the likelihood of the parameters β and γ under complete observation, as

$$\begin{array}{rl} \ell (\beta ,\gamma ) & =\int_{0}^{\tau }[\log\;\{\beta S(t)\bar{I}(t)\}dN(t)-\beta S(t)\bar{I}(t)dt+\log\;\{\gamma I(t)\}dR(t)-\gamma I(t)dt]. \end{array}$$

The first thing to notice is that the log-likelihood can be written as a sum of terms only depending on β and only on γ , respectively, implying that β and γ can be maximized separately. The MLE of β can then be analytically derived by taking the derivative of the part involving β , with respect to the parameter β , leading to (ignoring a $\beta^{-1}$ term)

(3) $$U(\beta )=\int_{0}^{\tau }\{dN(t)-\beta S(t)\bar{I}(t)dt\},$$

and setting this to zero. This yields

(4) $${\hat{\beta }}_{\mathrm{MLE}}=N(\tau )/\{\int_{0}^{\tau }S(t)\bar{I}(t)dt\}$$

as the MLE of β . Similarly (assuming R(0)=0 ), we arrive at

$${\hat{\gamma }}_{\mathrm{MLE}}=R(\tau )/\{\int_{0}^{\tau }I(t)dt\}.$$

These estimates can be readily calculated from the current data, leading to the values 1.988 and 0.509 for β and γ , respectively. Estimates of the variances of ${\hat{\beta }}_{\mathrm{MLE}}$ and ${\hat{\gamma }}_{\mathrm{MLE}}$ are also provided in Becker and Britton.^9^ From now on we concentrate on the transmission parameter β . We see from Becker and Britton^9^ that $\mathrm{se}({\hat{\beta }}_{\mathrm{MLE}})={\hat{\beta }}_{\mathrm{MLE}}/\sqrt{N(\tau )}$ . This gives 0.064 for the standard error of ${\hat{\beta }}_{\mathrm{MLE}}$ .

### 2.3. A convenient data format

Counting processes and their associated rates play a pivotal role in survival analysis so it is not surprising that methods from survival analysis can be used to estimate transmission parameters in SIR models. The rates of the counting processes defined above are all aggregated over all susceptible and infected individuals. The central contribution of this paper is to ‘disaggregate’ population counting processes into individual underlying data and structuring this information into a particular format often used in survival analysis with time-dependent covariates, which we refer to as *long format survival data* set. Restricting our attention to N(t) , which is counting the total number of new infections occurring in (0,t] , the link with survival analysis becomes clearer if we consider each of the individual counting processes $N_{i}(t)$ leading to $N(t)=\sum_{i=1}^{n}N_{i}(t)$ . Thus, $N_{i}(t)$ counts the number of new infections *of individual* i occurring in (0,t] , and this will be 1 or 0, depending on whether individual i has been infected or not before time t . Define $S_{i}(t)$ , $I_{i}(t)$ , and $R_{i}(t)$ to be random indicator variables taking the value 1 if at time t individual i is susceptible, infected or recovered, respectively, and 0 otherwise. The total number of susceptible, infected and recovered individuals is then $S(t)=\sum_{i=1}^{n}S_{i}(t)$ , $I(t)=\sum_{i=1}^{n}I_{i}(t)$ , and $R(t)=\sum_{i=1}^{n}R_{i}(t)$ , respectively. Individual i is only at risk of becoming infected while being susceptible. The usual notation in survival to indicate whether or not individual i is at risk at time t is $Y_{i}(t)$ . Since the event of interest here is becoming infected, being at risk is the same as being susceptible, for which we have already defined the notation $S_{i}(t)$ , so we have $Y_{i}(t)=S_{i}(t)$ . In the remainder of this paper we will be using $Y_{i}(t)$ when recalling general theory from survival analysis, and $S_{i}(t)$ when applying this to the SIR model. The underlying infection rate for subject i equals $\beta \bar{I}(t)$ . The rate of the *observed* counting process $N_{i}(t)$ equals $\beta \bar{I}(t)$ while being susceptible, which can then be written as $\beta S_{i}(t)\bar{I}(t)$ . In this way, adding over all individuals, we see that the rate of the observed aggregate counting process N(t) equals $\sum_{i=1}^{n}\beta S_{i}(t)\bar{I}(t)$ , leading to a total rate of $\beta S(t)\bar{I}(t)$ , the same as in Equation (2). Also, each *infected* individual has a constant rate γ of recovering.

This perspective is called *individual-based* or *agent-based* modelling, see KhudaBukhsh et al.^15^ With the purpose of estimating β , we can exploit the link to survival analysis, again under complete observation, by translating the original data, documenting the number of S, I, R individuals over time, into a long format survival data set where each susceptible subject occurs individually, and where at the same time the number of infected individuals is kept track of, using start-stop notation, also called Andersen-Gill^16^ or counting process notation.

The long format survival data for the first four susceptible individuals is shown in Table 1. The subject with id=1 is the first infection; this individual does not appear in the data, since he/she is no longer at risk for infection. The other id’s are sorted according to their time of infection. The follow-up time for id=5 is spread out over four follow-up intervals (Tstart, Tstop), over which the proportion of infectious individuals pinf is constant; the first, (0–0.323), with a single infected individual, the second, (0.323–1.03), with two infected individuals, etcetera. The status indicator equals 1 if the follow-up interval ends with an infection of the individual in question. This type of individual-level data format with follow-up intervals is routinely used with time-dependent covariates in survival analysis. Indeed, later we will use pinf, possibly transformed, as a time-dependent covariate.

**Table 1.** Long Format Survival Data for the First Four Susceptible Individuals.

id	w	Tstart	Tstop	status	pinf	fuptime
2	1	0	0.323	1	0.001	0.323
3	1	0	0.323	0	0.001	0.323
3	1	0.323	1.03	1	0.002	0.711
4	1	0	0.323	0	0.001	0.323
4	1	0.323	1.03	0	0.002	0.711
4	1	1.03	1.14	1	0.003	0.106
5	1	0	0.323	0	0.001	0.323
5	1	0.323	1.03	0	0.002	0.711
5	1	1.03	1.14	0	0.003	0.106
5	1	1.14	1.15	1	0.004	0.010

Each row represents a time interval for one individual. Variable ‘id’ is an identifier for the susceptible individuals, ‘w’ is a weight variable, ‘Tstart’ and ‘Tstop’ indicate start and end of time intervals over which the number of infecteds is constant, ‘status’ indicates whether the individual ‘id’ is infected (1) or not (0) at the end of the interval. The variable ‘pinf’ is the proportion of infected individuals during the time interval, and ‘fuptime’ is the length of the time interval.

A more concise version of this long format data can be obtained by combining all the rows with the same time interval (tstart, time), and adding all the weights, separately for status=0 and status=1, as shown in Table 2.

**Table 2.** Long Format Survival Data for the First Four Time Intervals.

w	Tstart	Tstop	status	pinf	fuptime
1	0	0.323	1	0.001	0.323
998	0	0.323	0	0.001	0.323
1	0.323	1.03	1	0.002	0.711
997	0.323	1.03	0	0.002	0.711
1	1.03	1.14	1	0.003	0.106
996	1.03	1.14	0	0.003	0.106
1	1.14	1.15	1	0.004	0.010
995	1.14	1.15	0	0.004	0.010

Each row represents a time interval for possibly multiple individuals (the number of which is indicated by ‘w’), over which the number of infected individuals is constant, ending with either an infection (‘status’ = 1) or not (‘status’ = 0).

## 3. Estimating the transmission parameter in the standard SIR model

This section has two aims. The first is to connect the known results in Sections 2.1 and 2.2 to standard survival analysis techniques and show that these known results can be recovered using long format survival data with routine techniques and standard software. The second aim is to show how novel extensions have become within reach, such as formal tests for time-invariance of β , and smooth estimation of β(t) , again using standard software.

### 3.1. Multiplicative models

#### 3.1.1. Cox models

Recall that the rate of $N_{i}(t)$ equals $\beta \bar{I}(t)$ while being at risk. The Cox model assumes that the rate of the event equals

$$\begin{array}{rl} & Y_{i}(t)h_{0}(t)\exp\;(\beta_{1}X_{i1}(t)+\cdots +\beta_{p}X_{ip}(t))=Y_{i}(t)h_{0}(t)\exp\;(\beta^{⊤}X_{i}(t)). \end{array}$$

Here $Y_{i}(t)$ again is the at risk indicator, $h_{0}(t)$ is an unspecified baseline hazard, $X_{i1}(t),\ldots ,X_{ip}(t)$ are possibly time-dependent covariates, and $\beta_{1},\ldots ,\beta_{p}$ are regression coefficients to be estimated. In the absence of ties, the vector of regression coefficients is estimated by maximizing the *partial likelihood*, given by

(5)

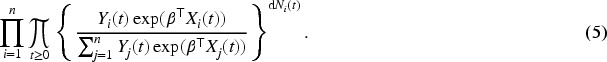


The product integral 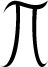 is a continuous version of a product, in the same way that an integral is a continuous version of a sum, see for instance Aalen et al.^17^ This partial likelihood is maximized with respect to β to obtain estimates $\hat{\beta }$ of β . For given β , in the absence of ties the estimate of the baseline hazard increment is given by Breslow’s estimate

(6) $${\hat{h}}_{0}(t)=\frac{dN(t)}{\sum_{i=1}^{n}Y_{i}(t)\exp\;(\beta^{⊤}X_{i}(t))},$$

with $N(t)=\sum_{i=1}^{n}N_{i}(t)$ . In the presence of ties, several methods for dealing with them have been developed and implemented in standard software. The formulas get more difficult, however, so we stick with (5) and (6).

If we take p=1 , $X_{i1}(t)=\log\;(\bar{I}(t))$ , and fix $\beta_{1}=1$ , we get a hazard rate of $h_{0}(t)\exp\;(\log\;(\bar{I}(t)))=h_{0}(t)\bar{I}(t)$ . Using Equation (6) we get

(7) $$\begin{array}{r} {\hat{h}}_{0}(t)=\frac{dN(t)}{\sum_{i=1}^{n}S_{i}(t)\exp\;(\log\;(\bar{I}(t)))}=\frac{dN(t)}{S(t)\bar{I}(t)}. \end{array}$$

**Figure 4.**
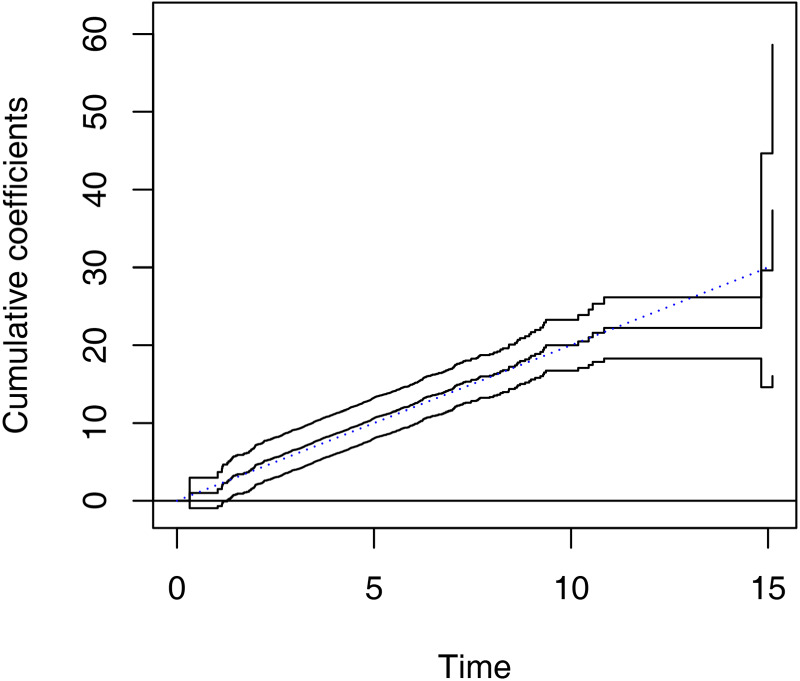
Estimated cumulative hazard over time; the solid lines denote the estimate and the pointwise 95% confidence intervals, while the dotted line represents the true cumulative hazard, equal to βt .

The resulting estimated cumulative hazard (cumulative transmission rate) is shown in Figure 4. It closely follows the true cumulative transmission rate, which equals βt , with β=2 .

If we fit a Cox model with $\log\;(\bar{I}(t))$ as an *offset* (meaning we are not estimating the associated regression coefficient, but setting it to one), then the baseline hazard rate of the Cox model becomes $h_{0}(t)\exp\;(\log\;(\bar{I}(t)))=h_{0}(t)\bar{I}(t)$ , the estimated baseline rate ${\hat{h}}_{0}(t)$ should equal $\hat{\beta }(t)$ , and the cumulative baseline hazard ${\hat{H}}_{0}(t)=\sum_{t_{i}\leq t}{\hat{h}}_{0}(t_{i})$ should resemble βt , in other words a straight line with intercept 0 and slope β . Fitting the Cox regression seems to recover the constant rate, although care is needed to extract the baseline hazard due to the offset term.

#### 3.1.2. Poisson regression

The Cox model uses an unspecified baseline hazard and does not use the assumption that the transmission rate is constant. We could attempt to estimate the transmission rate, assuming that it is constant, based on the well established epidemiological occurrence over exposure (O/E), see Clayton and Hills.^18^ The variance of the log rate is the inverse of the number of events, by which we could construct a 95% confidence interval of the estimated rate. In the data of Figure 2 we have a total of 974 events (new infections), while the exposure (total time at risk) is the sum of w times fuptime, which equals 490. This yields an O/E of 974/490, which is 1.988. The estimate is quite close to the true rate 2, and also identical to the ${\hat{\beta }}_{\mathrm{LME}}$ obtained by the direct formula of Becker and Britton.^9^ The variance of the logarithm of this rate equals 1/974, by which we obtain a 95% confidence interval of the log rate of 0.62–0.75, and, taking exponentials, a 95% confidence interval for the transmission rate itself of 1.87–2.12.

Alternatively, Poisson regression can be used. The expected number of infections in a short interval of length Δt around t equals $\beta \bar{I}(t)\Delta t$ , so with a log link this equals $\exp\;(\log\;\beta +\log\;\bar{I}(t)+\log\;\Delta t)$ . This means that fitting a GLM Poisson model with log link, and with both $\log\;\bar{I}(t)$ and logΔt as offset terms, we obtain an estimate of logβ . Taking the exponent of the estimate of logβ then provides an estimate of β , which again gives exactly the same result for $\hat{\beta }$ as Becker and Britton.^9^ The standard errors from the Poisson regression and the occurrence/exposure formulas are also exactly the same.

So far, this shows that known results, in particular the MLE of Becker and Britton,^9^ can be exactly replicated using the long format survival data and subsequently using routine (Poisson GLM) techniques with standard software. But we can go one step further. A novel extension is to use the Poisson regression model to estimate β(t) as a smooth function of time using splines. We use the natural splines (cubic splines that are linear beyond the outermost knots), as implemented in the Ns() function in the {Epi} package, but other splines can also be used.

**Figure 5.**
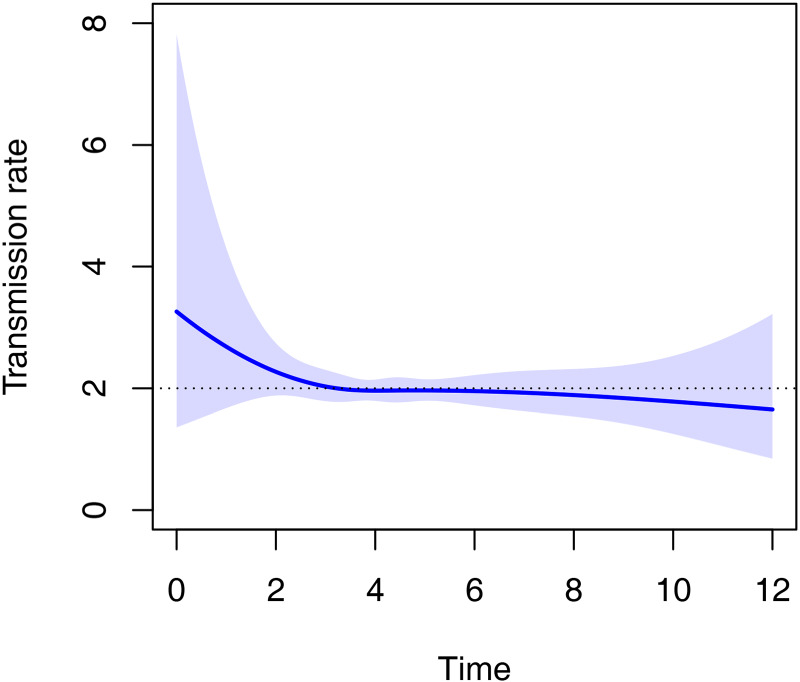
Smooth estimate of the transmission rate β(t) (solid line, 95% confidence limits in shaded area).

Figure 5 shows a plot of the spline-based estimate of β(t) , using matshade() from the {Epi} package. It can be seen that the curve is more or less constant, with wider confidence intervals in the beginning and at the end. Note that the use of splines in combination with Poisson modelling has been used before in infectious disease modelling,^19^ to estimate time-varying transmission and removal rates. There the Poisson model was used to model the counts of observed infecteds and removed individuals in discrete time intervals, rather than the underlying intensity functions of the relevant counting processes of (2).

### 3.2. Additive hazards

The additive hazards model^20,21^ specifies the rate of new infections as a sum of (typically time-dependent) linear combinations of the covariates, which themselves may also be time-dependent:

(8) $$\lambda_{i}(t)=Y_{i}(t)\{\beta_{0}(t)+\beta_{1}(t)X_{i1}(t)+\cdots +\beta_{p}(t)X_{ip}(t)\}.$$

The first term within brackets, $\beta_{0}(t)$ , is an intercept, comparable to the baseline hazard in the Cox model, $X_{i1}(t),\ldots ,X_{ip}(t)$ is a set of possibly time-dependent covariates, and $\beta_{1}(t),\ldots ,\beta_{p}(t)$ are the regression coefficients. Typically these are taken to be time-dependent, but we will also consider the case later where the β(t) ’s are constant over time. Aalen’s ordinary least squares (OLSs) estimates focus on the cumulative regression functions $B_{j}(t)=\int_{0}^{t}\beta_{j}(s)ds$ .

Defining vectors $N(t)=(N_{1}(t),\ldots ,N_{n}(t){)}^{⊤}$ , $B(t)=(B_{0}(t),B_{1}(t),\ldots ,B_{p}(t){)}^{⊤}$ , and the matrix X(t) , with i th row $\left(Y_{i}(t),Y_{i}(t)X_{i1}(t),\ldots ,Y_{i}(t)X_{ip}(t)\right)$ , then Aalen et al.^17^ derive

(9) $$\text{d}\hat{B}(t)=(X{\left(t\right)}^{⊤}X(t){)}^{-1}X{\left(t\right)}^{⊤}\text{d}N(t)$$

as estimate of the increment of B(t) , provided X(t) has full rank. In the absence of the intercept term $\beta_{0}(t)$ in the model, the constant elements in the first column of X(t) are removed.

Applying this model to the SIR setting, since the rate of each susceptible individual equals $\beta \bar{I}(t)$ , we can view this as a term β/n being added to the hazard for each infectious individual. So we would have no intercept, p=1 , and $X_{i1}(t)=X_{i}(t)=\bar{I}(t)$ for each individual, and X(t) would be a n×1 vector with i th element $Y_{i}(t)X_{i}(t)=S_{i}(t)\bar{I}(t)$ . Using Equation (9) leads to

(10) $$\begin{array}{rl} \hat{\beta }(t) & ={\left\{\sum_{i=1}^{n}(S_{i}(t)\bar{I}(t){)}^{2}\right\}}^{-1}\sum_{i=1}^{n}S_{i}(t)\bar{I}(t)dN_{i}(t)\\ & =\frac{\bar{I}(t)dN(t)}{S(t)\{\bar{I}(t){\}}^{2}}=\frac{dN(t)}{S(t)\bar{I}(t)}. \end{array}$$

Here we have used that $S_{i}^{2}(t)=S_{i}(t)$ and $S_{i}(t)dN_{i}(t)=dN_{i}(t)$ (because an event can only happen when someone is at risk). Note that $\hat{\beta }(t)$ from Equation (10) corresponds with the estimated baseline hazard from Equation (7) in the Cox model. This $\hat{\beta }(t)$ can be estimated in the {timereg} package in R, by fitting an additive hazards model with $\bar{I}(t)$ (in column pinf) as covariate, *excluding* an intercept. This idea has been used before in Wolkewitz et al.^22^ The result is as shown in Figure 4, pointwise 95% confidence interval are obtained with the {timereg} package.

It is reassuring that the estimate of B(t) indeed seems to follow a straight line, indicating constant β(t) . The hypothesis of β(t) being constant can be tested in the {timereg} package, yielding p-values of 0.72, both for the Kolmogorov–Smirnov and the Cramer–von Mises tests. For background on these tests we refer to Martinussen and Scheike.^23^ This again is a novel extension of the toolkit. The slope is approximately equal to β=2 , as we would hope, see the dotted line in Figure 4.

Inspired by least squares theory, a least squares estimator would be given by a variant of the displayed equation between (2.6) and (2.7) of Lin and Ying,^24^ the difference being the absence of the baseline hazard term, given by

$$U(\beta )=\sum_{i=1}^{n}\int_{0}^{\tau }X_{i}(t)\left\{dN_{i}(t)-Y_{i}(t)\beta X_{i}(t)dt\right\},$$

where we recall that the time-dependent covariate is given by $X_{i}(t)=\bar{I}(t)$ , and $Y_{i}(t)$ is the at risk indicator, which in our case was earlier denoted by $S_{i}(t)$ . We will first elaborate on the general theory, then replace $X_{i}(t)$ by $\bar{I}(t)$ and $Y_{i}(t)$ by $S_{i}(t)$ to apply it to the SIR case. Setting U(β) to zero and solving for β yields

$${\hat{\beta }}_{\mathrm{OLS}}=\frac{\sum_{i=1}^{n}\int_{0}^{\tau }X_{i}(t)dN_{i}(t)}{\sum_{i=1}^{n}\int_{0}^{\tau }Y_{i}(t)X_{i}^{2}(t)dt}.$$

Following Lin and Ying,^24^ define

$$\begin{array}{rl} A & =n^{-1}\sum_{i=1}^{n}\int_{0}^{\tau }Y_{i}(t)X_{i}^{2}(t)dt,B=n^{-1}\sum_{i=1}^{n}\int_{0}^{\tau }X_{i}^{2}(t)dN_{i}(t). \end{array}$$

Then the variance of ${\hat{\beta }}_{\mathrm{OLS}}$ can be consistently estimated by $B/(nA^{2})$ , which equals

$$\frac{\sum_{i=1}^{n}\int_{0}^{\tau }X_{i}^{2}(t)dN_{i}(t)}{{\left(\sum_{i=1}^{n}\int_{0}^{\tau }Y_{i}(t)X_{i}^{2}(t)dt\right)}^{2}}.$$

We go back to the formulas for ${\hat{\beta }}_{\mathrm{OLS}}$ and its variance, and now replace $X_{i}(t)$ by $\bar{I}(t)$ and $Y_{i}(t)$ by $S_{i}(t)$ , leading to

(11) $${\hat{\beta }}_{\mathrm{OLS}}=\frac{\int_{0}^{\tau }\bar{I}(t)dN(t)}{\int_{0}^{\tau }\{\bar{I}(t){\}}^{2}S(t)dt}.$$

Note first that ${\hat{\beta }}_{\mathrm{OLS}}$ is of a similar form as the estimate $\hat{\beta }(t)=\frac{\bar{I}(t)dN(t)}{\left\{\bar{I}(t){\}}^{2}S(t\right)}$ of Equation (10), but with both the numerator and denominator being integrated over time. Note also that ${\hat{\beta }}_{\mathrm{OLS}}$ is similar to ${\hat{\beta }}_{\mathrm{MLE}}$ in Equation (4), except that ${\hat{\beta }}_{\mathrm{OLS}}$ has an extra weighting term $\bar{I}(t)$ in both numerator and denominator.

The estimate of its variance is given by

(12) $$\frac{\int_{0}^{\tau }\{\bar{I}(t){\}}^{2}dN(t)}{{\left(\int_{0}^{\tau }\{\bar{I}(t){\}}^{2}S(t)dt\right)}^{2}}.$$

Applying Equations (11) and (12) to the data of Figure 2 gives ${\hat{\beta }}_{\mathrm{OLS}}=1.97$ , with an estimated standard error of 0.070, which is about 10% larger than the standard error of ${\hat{\beta }}_{\mathrm{MLE}}$ .

## 4. Relaxing assumptions about the epidemic model

So far we saw that for estimating β , when not assuming β to be constant, the estimate of β(t) obtained from the additive hazards model and the Cox model gave exactly the same result. The perspective of the additive hazards model is that we can view the rate $\beta \bar{I}(t)$ of a susceptible individual as additive in the number of infected individuals at time t ; each infected individual *adds* β/n to the hazard. In contrast, the multiplicative hazards model views $\log\;(\bar{I}(t))$ as a multiplicative term, with regression coefficient fixed to one (an offset term).

Without any other covariates both perspectives are equally valuable and they give the same result when β(t) is estimated non-parametrically as a time-dependent function, and slightly different but quite comparable results when estimating a time-constant β . In Section 4.1 we discuss the incorporation of interventions, which in some cases can be conveniently modelled with multiplicative models like the Cox model or Poisson regression. In Section 4.2 we discuss population structures, which are naturally modelled using the additive hazards model. Both elements can be combined using hybrid models like the Cox-Aalen modelled, as argued in Section 4.3. Section 4.4 discusses the issue of unobserved heterogeneity in susceptibility to infection.

### 4.1. Incorporating interventions

Implementations of intervention measures are often most naturally expressed in a multiplicative way. It makes sense to assume that such measures will decrease the hazard of every individual by a certain percentage. This is the case for non-pharmaceutical interventions such as mask wearing, keeping physical distance and lock-down measures. Other non-pharmaceutical interventions, such as case isolation and contact tracing, act by effectively reducing the infective period. Vaccination, a pharmaceutical intervention, acts by depleting the pool of susceptibles. We will consider only the first class of non-pharmaceutical interventions for which it is reasonable to state that it acts in a multiplicative way on the transmission parameter. In ideal settings the effect of the intervention can be estimated together with the transmission parameter, using Cox or Poisson regression.

**Figure 6.**
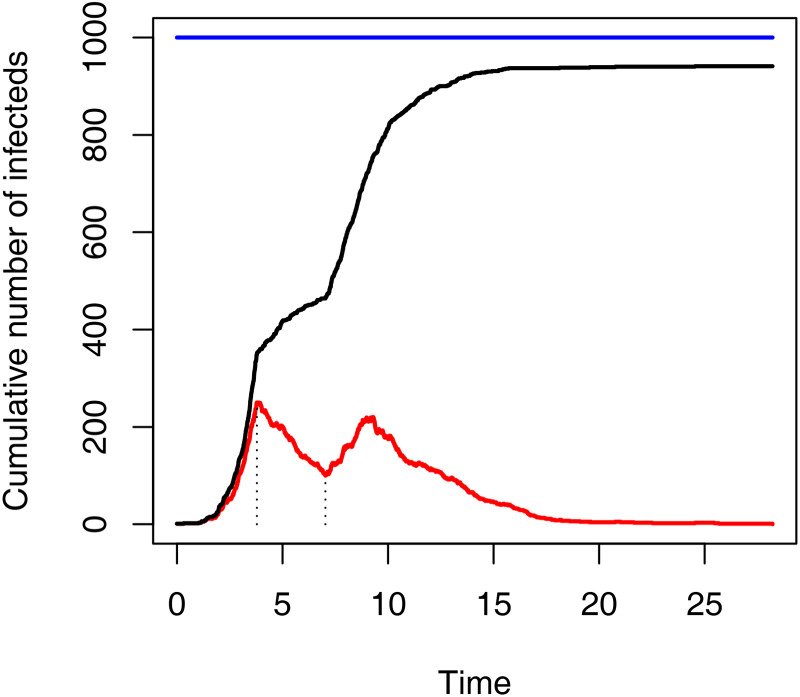
Stacked plot showing the number of susceptible, infected and recovered individuals over time, with intervention start and end indicated.

We are now going to explore the use of Cox and Poisson regression models in a situation where intervention measures are put into place. In the following simulation, the intervention is triggered by the proportion of infected individuals reaching a certain threshold. Figure 6 shows the result of a simulated outbreak according to an SIR model, where an intervention is started as soon as 25% of the population is infected, and suspended as soon as that percentage has dropped to 10%. The effect of the intervention is 80%, in that the original transmission parameter β is 2, and after intervention it is 2⋅0.2=0.4 . The time points where the intervention is implemented (when 25% of the population is infected) and stopped (when 10% of the population is infected) are indicated at the bottom.

We see that after the intervention has been suspended the number of infections rises again, almost to 25%, but it decreases again just after that. This time 59 subjects that were at risk for infection never got infected during the epidemic. In the data it occurs as one subject with weight w=59 .

**Figure 7.**
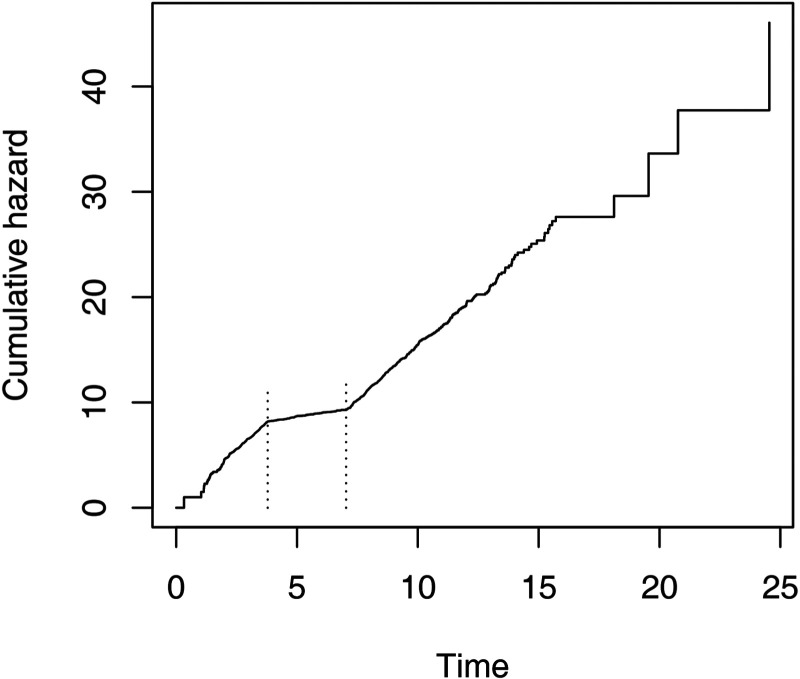
Estimated baseline hazard of the Cox model under intervention.

We can pick up the changes in transmission rates without knowing the times at which the interventions were in effect. Our first attempt is with a non-parametric estimate $\hat{B}(t)$ of the cumulative rate, using coxph(), shown in Figure 7. As in Figure 2, the slope of the cumulative rate $\hat{B}(t)$ gives an idea about the transmission rate β(t) .

Knowing the time of the intervention, we can see an initial slope of about 2 before the intervention, followed by a decreasing rate of infection can be seen, including an increase to the rate before intervention after the intervention has been suspended. We can also use Poisson regression again, with natural splines (same choice of knots as before, based on quartiles), to obtain a smooth estimate of β(t) (note, however, that the true β(t) is piecewise constant, not smooth).

**Figure 8.**
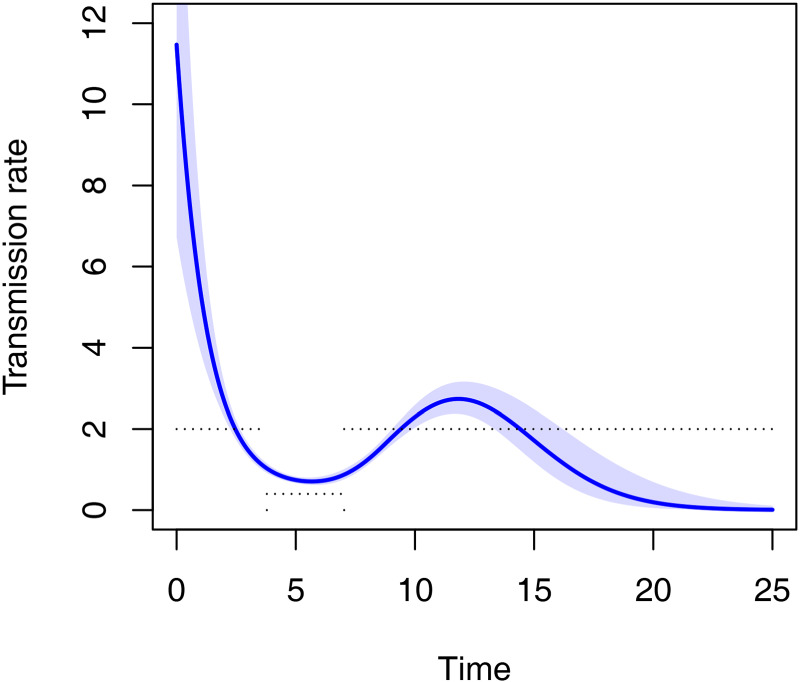
Estimated time course of transmission rate using Poisson regression, with 95% confidence intervals; the true transmission rate is indicated with dotted lines.

This time some of the non-constant effects of the splines are significant, pointing towards a non-constant transmission rate. Figure 8 shows a plot of the estimated transmission rate β(t) . The true β(t) is shown as dotted lines. Because the natural splines try to impose a smooth line through a time-dependent rate that is inherently piecewise constant, the fitted curve is smoothly decreasing from above β=2 to about 2*0.2=0.4 , and back to β=2 .

To be able to study the effect of the interventions, knowing when they were put in place and later suspended, we have to add information about the interventions to the data. Estimates of the (assumed constant) transmission rate can be estimated with the epidemiological occurrence to exposure, as in Subsection 3.2.2, this time by intervention period. The O/E numbers by intervention period are 349/166=2.10 , 115/329=0.35 and 476/235=2.02 , for pre-intervention, intervention and post-intervention periods, respectively. These estimated rates correspond reasonably to the true transmission rates of 2, 0.4 and 2 in the three periods.

A weighted Cox regression with intervention as categorical covariate (and logarithm of number of infected individual as offset), gives NA’s for estimates and standard errors (not shown). This is because the intervention is applied for all subjects in the same period, and hence the intervention effect is confounded with the baseline hazard. The intervention effect would be identifiable if different subjects would experience the interventions at different points in time.

The intervention effect is also identifiable with parametric restrictions on the baseline hazard, like a piecewise constant assumption. These can be retrieved using weighted Poisson regression, again with log of interval time and log of proportion of infected individuals as intercept, and intervention as categorical covariate. Results are not shown here for brevity, but they are consistent with the O/E approach, and can be found in the accompanying online version on https://github.com/survival-lumc/SIRsurvival.

### 4.2. Incorporating population structure

An interesting application where additive hazards occur very naturally is one where the population can be sub-divided into groups, such as age groups, occupation, households, schools, for instance, and where infections can occur between susceptibles and infected individuals within the same group or across groups.

**Figure 9.**
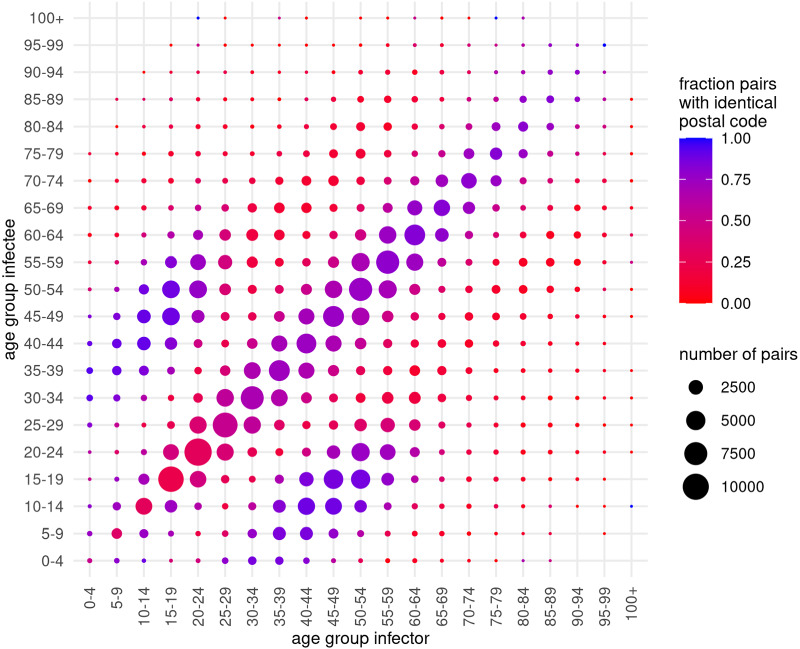
Pairs of notified cases that transmitted infection, by age of infector and of infectee, across age groups until March 2022 in the Netherlands.

Figure 9 below shows the number of pairs of reported cases of COVID-19 that transmitted infection across different age groups in the Netherlands until March 2022. It nicely shows the number of transmissions are highest across similar age groups and after that across age groups differing by one generation (most probably transmissions within the same household). But they are numbers, some of which could be higher or lower simply because the group sizes are different. The objective would be to estimate the transmission rates across age groups.

We thus consider multiple groups (for instance age groups) that can infect each other. For group j=1,…,J define $S_{j}(t)$ , $I_{j}(t)$ , $R_{j}(t)$ to be the total number of susceptible, infected and recovered individuals in group j at time t , and denote the total number of subjects in group j by $n_{j}=S_{j}(t)+I_{j}(t)+R_{j}(t)$ . We again assume that the groups are closed, that is no migration, no births or deaths.

Within group j , new infections happen with rate $S_{j}(t)\sum_{k=1}^{J}\beta_{kj}(t){\bar{I}}_{k}(t)$ , with ${\bar{I}}_{k}(t)=I_{k}(t)/n_{j}$ . The idea is that each of the susceptibles in group j at time t , of which there are $S_{j}(t)$ , can be infected by an infectious individual from within group j itself or from within one of the other groups. The (potentially time-varying) transmission rate parameter $\beta_{kj}(t)$ describes the intensity at which contacts are made between an infected individual from group k and a susceptible individual from group j leading to a new infection. For respiratory infections such as COVID-19, when we have a matrix with contact rates we would expect reciprocity of contacts for most infections (if I contact you, you contact me). If such contacts would equally likely lead to infection, by knowing $\beta_{jk}$ and population sizes, we know the value of $\beta_{kj}$ . It is not clear whether this is the case here. Finally we define the counting processes $N_{j}(t)$ , j=1,…,J , counting the total number of susceptibles in group j becoming infected within (0,t] .

Interest is in estimating the (possibly time-varying) transmission parameters $\beta_{kj}(t)$ .

Again we can look from the individual perspective, define a counting process for each individual i from each group j , $N_{ji}(t)$ , having rate $Y_{ji}(t)\sum_{k=1}^{J}\beta_{kj}(t){\bar{I}}_{k}(t)$ , where ${\bar{I}}_{k}(t)=I_{k}(t)/n_{j}$ , with $n_{j}$ the size of group j . This is an *additive hazards model* without intercept, and J time-dependent covariates ${\bar{I}}_{k}(t)$ , where $Y_{ji}(t)$ is the at risk indicator of subject i in group j for being susceptible to infection.

To simplify notation, consider one group j of susceptibles. We will fix that group, suppress j in the notation everywhere, and let n be the size of that group. Within this group, individual i has rate

(13) $$\lambda_{i}(t)=Y_{i}(t)\sum_{k=1}^{J}\beta_{k}(t){\bar{I}}_{k}(t).$$

This rate conforms to the additive hazards model with rate $Y_{i}(t)\{\beta_{0}(t)+\sum_{k=1}^{J}\beta_{k}(t)X_{ik}(t)\}$ , as in Equation (8), with two non-standard aspects. The first is that there is no intercept $\beta_{0}(t)$ , the second is that for each time point t the time-dependent covariates $X_{ik}(t)$ do not depend on i , that is they are the same for all susceptible individuals. This has the important implication that the k th column of the matrix X(t) used in Equation (9) is of the form $Y(t){\bar{I}}_{k}(t)$ , $Y(t)=(Y_{1}(t),\ldots ,Y_{n}(t){)}^{⊤}$ . As a result X(t) is of rank 1, which implies that the $\beta_{k}(t)$ ’s are not identifiable from the data when the $\beta_{k}(t)$ ’s are allowed to vary freely. To be able to estimate the $\beta_{k}(t)$ ’s one would need some kind of smoothing or restricting the $\beta_{k}(t)$ ’s to be constant or piecewise constant on time intervals. The OLS approach that is commonly used in additive hazards will probably not constrain the estimated transmission parameters ${\hat{\beta }}_{kj}$ to be non-negative. It would be of interest to estimate these parameters under a non-negativity constraint, as pursued in Lu et al.^25^

### 4.3. Hybrid (Cox-Aalen) models

As we have seen, the effect of an intervention is most naturally incorporated as a multiplicative effect. The same goes for the effect of measured characteristics of the susceptible individuals, like gender, or perhaps known risk factors for infection. If we want to incorporate such effects multiplicatively and additionally have a structured population that would call for an additive hazards structure, then hybrid models would be of interest where the hazard of subject i in group j takes the form

$$\lambda_{ji}(t)=\exp\;(\gamma^{⊤}X_{ji})\sum_{k=1}^{J}\beta_{kj}{\bar{I}}_{k}(t),$$

with $X_{ji}$ a vector of baseline covariates of subject i in group j would be of interest. This type of model is known as a Cox-Aalen model, and has been studied by Scheike and Zhang.^26^ We will not pursue this method in this paper.

### 4.4. Heterogeneity in susceptibility to infection

It is a huge simplification to assume that each susceptible individual is equally susceptible to becoming infected, or that each infected individual is equally likely to infect others. With individual knowledge of covariates, these could be incorporated into the survival analysis models, be they additive hazards, Cox or Poisson models. In the absence of such information, a natural extension to the models considered is to add individual random effects expressing such heterogeneity. In survival analysis, models incorporating such random effects are known under the term *frailty models*, see for instance.^27^ It is possible to fit such frailty models also for SIR models.

**Figure 10.**
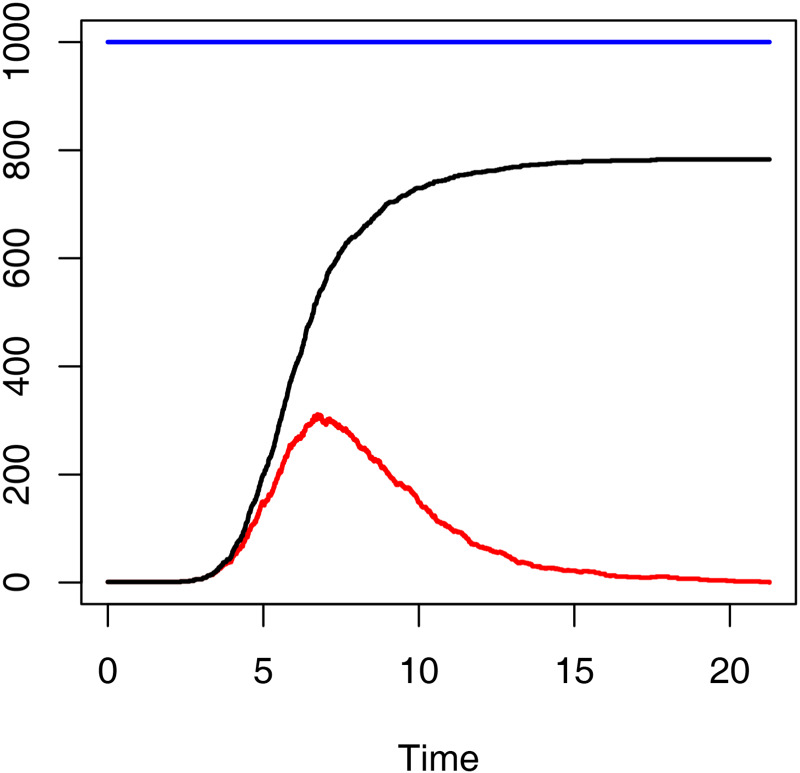
Stacked plot showing the number of susceptible, infected and recovered individuals over time from a stochastic susceptible-infectious-recovered (SIR) model with heterogeneity in susceptibility.

Figure 10 shows the result of a simulated outbreak, based on the same parameters as Figure 2, but where the transmission parameter associated with a susceptible individual equals βZ , with Z a gamma random variable with mean one and variance $\sigma_{Z}^{2}=1$ . Thus, the mean transmission parameter over the population of susceptibles equals β , but susceptible individuals differ in their degree of susceptibility to infection.

This heterogeneity in susceptibility leads to an epidemic with considerably fewer infections compared to one with the same β and γ and no such heterogeneity. In fact, it can be shown by Jensen’s inequality that for models with unobserved heterogeneity, the assumption that all individuals are equally susceptible leads to an upper bound for the final proportion infected, see for instance.^28,29^

**Figure 11.**
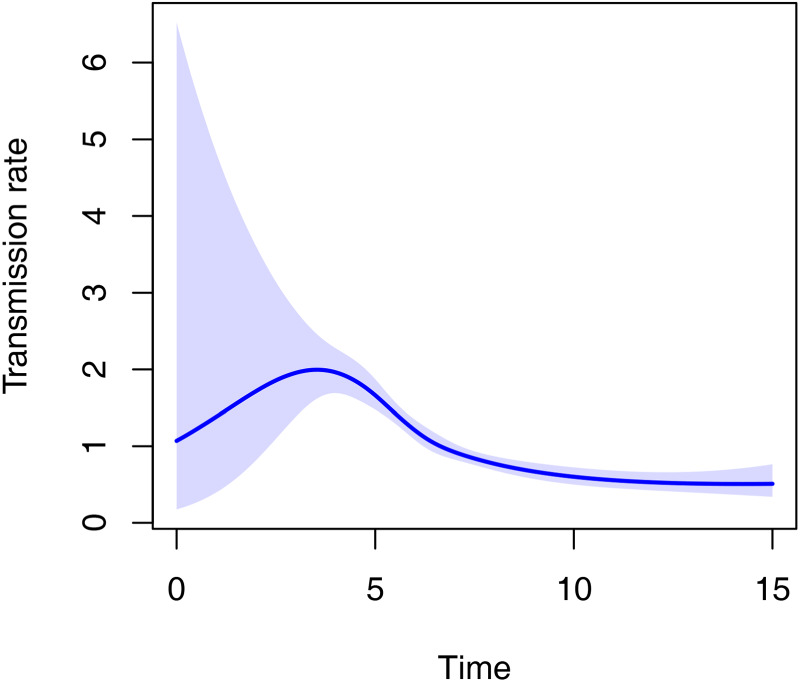
Estimate of β(t) with 95% confidence region, obtained using Poisson regression with cubic splines from an susceptible-infectious-recovered (SIR) model with heterogeneity in susceptibility.

If we estimate β from this generated data using maximum likelihood, for instance using Poisson regression as in Subsection 3.1.2, assuming it is time constant, we obtain an estimated β of 1.08 (95% confidence interval: 1.00–1.16). This is considerably lower than the true value 2, caused by the fact that the most susceptible individuals get infected first, resulting in less susceptible individuals remaining in the susceptible pool over time. As in standard survival analysis, the presence of the frailty terms induces time-varying behaviour of β , see Balan and Putter.^27^ This is indeed picked up using Poisson regression with splines, where we indeed see non-constant β(t) in Figure 11. The message is that if we do not account for heterogeneity in susceptibility we obtain time-varying estimates for the transmission rate, even though the actual transmission rate was constant, and we obtain a β(t) which is lower than the average of βZ over the distribution of Z at the onset of the epidemic.

Estimating β while incorporating the presence of unobserved heterogeneity in susceptibility could be done using an expectation-maximization (EM)-algorithm, but this is not pursued here.

## 5. Relaxing assumptions about the data

The type of data that we considered in this paper is too idealized in the sense that we typically do not know the time of infections exactly and we do not know the number of infectious and susceptible individuals exactly. Numbers of infections are usually aggregated over days or weeks. Moreover they are typically reported with some delay and they are incomplete. Methodology needs to be extended to deal with these more realistic data settings. We will not cover all of these aspects here, but we will show how the methods in this paper can still be used to estimate the transmission parameter β in case the (correct) probability distribution of the time to recovery after infection (the so-called infectivity kernel) is known, and aggregate data on the daily number of newly infected individuals is reported, as was the case for the COVID-19 infection. For now we assume there is no reporting delay or incompleteness.

We start from the fully observed data used before. To make it roughly in line with the recent COVID-19 epidemic, based on Figure 2 it seems reasonable to say that the time unit there is months, and that we have daily updates of the number of new infections. Let us say that after 10 infections the outbreak is ‘detected’ (at day 41), and that we set this to day 0 and start reporting daily new infections after that. That would give 1 new infection on days 1, 2, 3 and 4 within the first week of the outbreak (and many more after that).

Using the information on time to recovery, each newly infected individual on day d will be counted as an infectious individual on day d+j with probability $p_{j}=P(\mathrm{time}\;\mathrm{to}\;\mathrm{recovery}\geq j)$ . The expected number of infectious individuals on day $\tilde{d}$ then is a sum of the number of newly infected individuals on day $\tilde{d}-j$ , weighted by $p_{j}$ . We can then create a daily analysis data set where each day is represented by a number of individuals becoming infected with status = 1 (the number of newly infected individuals on that day), and a number of individuals that are susceptible and did not get infected on that day with status = 0 (the population size n minus the cumulative number of infected individuals until and including that day).

Table 3 shows the resulting long format survival data for the first week of the outbreak. Day 3 for instance has Tstart=2 and Tstop=3. Note that on days 4 through 7 no new infections occurred (after day 8 again), we therefore see no rows on these days with status = 1.

**Table 3.** Aggregate Daily Data of the First Week in Counting Process Format.

Tstart	Tstop	ninf	status	w
0	1	10.8	1	1
0	1	10.8	0	988
1	2	11.7	1	1
1	2	11.7	0	987
2	3	12.5	1	1
2	3	12.5	0	986
3	4	13.3	0	986
4	5	13.0	0	986
5	6	12.8	0	986
6	7	12.6	0	986

**Figure 12.**
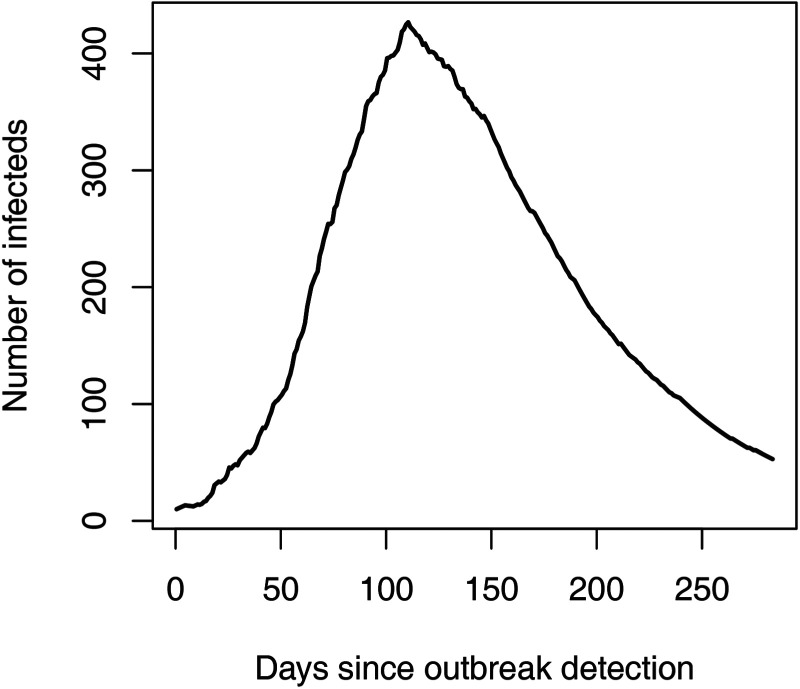
Expected number of infecteds over time.

Figure 12 shows the expected number of infected individuals over time (in days since the outbreak was detected).

After calculating pinf, the proportion of infected individuals, and fuptime, the time between the start and end of the reporting time period, which was one day, 1/30 of a month, we can then use Poisson regression with log link and logpinf and logfuptime as offsets, to obtain an estimate of β . It results in an estimated transmission parameter β of 1.97 (95% confidence interval 1.85–2.10).

The result again is very close to the true value of β=2 . The Poisson regression also gives a confidence interval. Note, however, that the estimate of the standard error of $\hat{\beta }$ is too optimistic, for two reasons. First, assuming known time-to-recovery distribution, the randomness in the actual time-to-recovery is ignored and replaced by their expectations. Second, the time-to-recovery distribution is typically estimated with considerable uncertainty in itself. These sources of randomness need to be taken into account to obtain correct standard errors and confidence intervals. This is outside the scope of this manuscript.

## 6. Application

**Figure 13.**
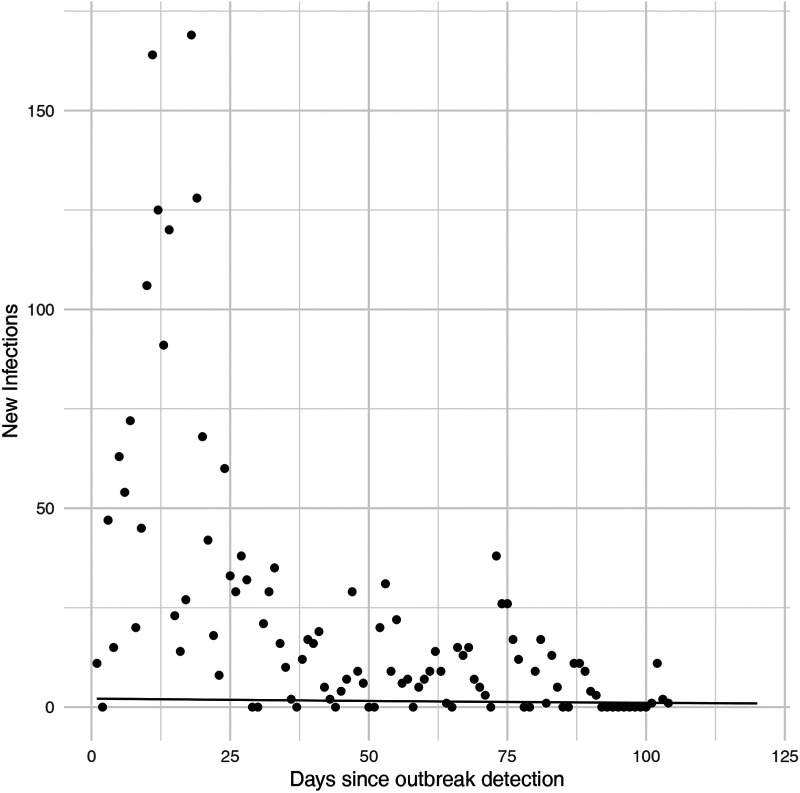
Daily counts of new infections, H1N1 outbreak on WSU campus 2009; the smooth curve represents the model-based fit based on the SIR model with 11 initially infected individuals. SIR: susceptible-infectious-recovered; WSU: Washington State University.

We consider data of an outbreak of H1N1 influenza (swine flu) on the campus of Washington State University (WSU) in the Fall of 2009.^15,30–33^ The aim of this re-analysis of the same data is to show how the techniques developed in this paper may be applied in practical settings where it is unclear how well the assumptions of the model are satisfied. Earlier accounts already argued that this data set may be considered as obtained from an approximately closed population, and under- or over-reporting of infections may be considered a relatively minor issue.^33^ The data consist of daily counts of new infections, which were kindly provided by dr. KhudaBukhsh, see Figure 6 of KhudaBukhsh et al.^15^ These are shown as the points in Figure 13.

Now, assuming, as in Schwartz et al.,^31^ an exponential time to recovery with rate γ=0.2 , and a population size n=18,234 , we can build a long format survival data set along the lines of Section 5. Poisson regression leads to an estimate of β of 0.195 (95% confidence interval 0.187–0.203). Schwartz et al.^31^ noticed a discrepancy of the newly infected individuals from the original data, as compared with that predicted by the model, see their Figure 2. We can indeed confirm that, as shown in Figure 13, where the original counts are plotted together with the model-based numbers of new infections. The reason for the immense lack of fit is the overall estimate $\hat{\beta }=0.195$ , which is smaller than the assumed γ=0.2 , leading to an $R_{0}<1$ , and hence to an immediate and persistent decrease of the ODE-based number of new infections over time.

**Figure 14.**
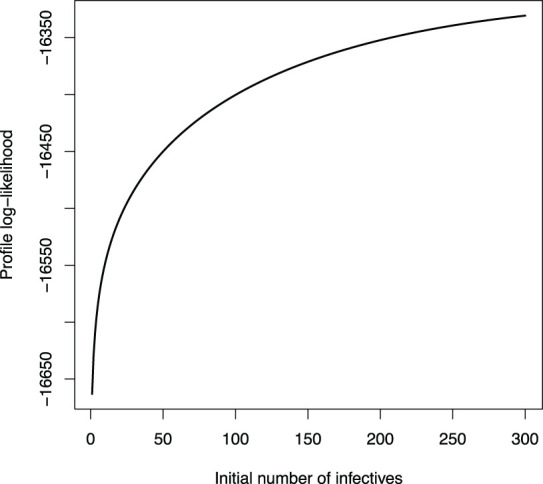
Profile log-likelihood, as a function of initial number of infectives.

Schwartz et al.^31^ went on to refit the model assuming a different number of initially infected individuals I(0) , using least squares estimation (LSE). We will follow the idea of re-estimating β with different number of initial infectives, but take a different approach by relying on a profile likelihood framework, profiling out I(0) . Concretely, for each value of I(0) in a reasonable range – we take I(0)=1,…,300 – we re-estimate β and calculate the maximized log-likelihood. Figure 14 below shows a plot of the maximized log-likelihood, as a function of I(0) , the initial number of infectives. The estimated transmission parameters decreased minimally from 0.196 to 0.175, as I(0) increased from 1 to 300. As can be seen from Figure 14, we cannot confirm the findings of Schwartz et al.^31^ based on LSE, which pointed to an optimal I(0) of 105. In fact, this choice would lead to an ODE-model-based number of new infections that also strictly decreases over time. An initial number of infectives of 300 would still lead to a strictly deasing number of newly infectives. See https://github.com/survival-lumc/SIRsurvival for code and output of these results.

**Figure 15.**
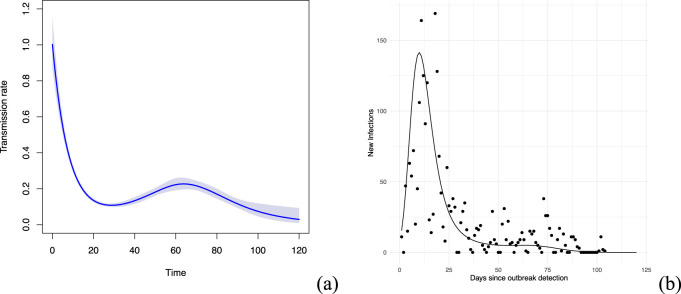
(a) Estimated time-varying transmission parameter, obtained from Poisson GLM with natural splines; (b) Daily counts of new infections, together with model-based numbers based on susceptible-infectious-recovered (SIR) model with time-varying β(t) .

The considerable lack of fit to the number of newly infected individuals seems to point towards violations of the original assumptions. Without aiming for a comprehensive assessment of all assumptions (exponential distribution of recovery with mean 5, γ=0.2 , the sample size, closed population, no mis-reporting), one assumption that is open for scrutiny is the assumption of constant transmission parameter β . In fact, based on the long format data that has already been constructed, it is rather straightforward to obtain a spline-based estimate of a time-varying transmission parameter β(t) , using Poisson regression, as illustrated in Subsection 3.1. This leads to the plot shown in Figure 15(a), suggesting a highly variable transmission parameter. This time-varying estimate of β(t) does provide a much better fit to the daily counts of newly infected individuals from the data, as can be seen in Figure 15(b), and at least explains the initial peak after about ten days and also the temporary increase about 60–70 days into the epidemic.

Note that other possible violations of the assumptions are also possible. In particular the assumption of a sample size of 18,000 has been discussed as well.^15^

## 7. Discussion

In this paper we have shown how standard methods from survival analysis can be used to estimate pivotal quantities in SIR models. In particular we have focussed on estimating the transmission parameter in the SIR model. We have illustrated the use of multiplicative models like the Cox model and Poisson regression, and of the additive hazards model, and we have argued for the usefulness of the Cox-Aalen model, which is a hybrid of multiplicative and additive models. The possibility of using these standard models with the wide availability of software to elucidate underlying pivotal parameters opens possibilities in many situations, for instance in structured and/or clustered data.

The work we presented here complements earlier work^34–36^ that takes a transmission network as a starting point, in part to avoid assuming some form of mass action, which is the basis of SIR-type models. We have taken a particular form of mass action as a starting point in order to avoid having to assume who infected whom. In our approach correct knowledge of the number of susceptible and infectious individuals over time is needed. In actual situations this knowledge is not readily available and needs to be further estimated from the available data and assumptions.

Clearly, more work is needed to take full advantage of survival analysis techniques in infectious diseases. First and most importantly, the data challenges need to be addressed more carefully. Issues like incompleteness of reporting of infections, reporting delay will severely complicate reliable knowledge of the number of infected and infectious individuals over time. Reporting delay is probably relatively easy to incorporate. Expanding on the discussion of Section 5, stating that each newly infected individual on day d will be counted as an infectious individual on day d+j with probability $p_{j}=P(\mathrm{time}\;\mathrm{to}\;\mathrm{recovery}\geq j)$ , we could say instead that each newly *reported* individual on day d is expected to be infected on day d−k , with a certain probability $\pi_{k}$ , expressing the distribution of reporting delay. Reporting incompleteness could be incorporated through a latent reporting indicator and a subsequent EM algorithm or a Bayesian approach.^15^ This would be a useful avenue for future work. Second, the issue of heterogeneity in susceptibility is of major interest. Using frailty models seems a very promising way of estimating the extent of variability in susceptibility in the population. This work is currently under development. Third, while multiplicative models have already been used to estimate the effect of interventions in slowing down the spread of an infection, additive hazards models have to the best of our knowledge not been used in infectious disease models. Finally, the use of hybrid multiplicative – additive models such as the Cox-Aalen model seems particularly attractive.
